# Type 1 Diabetes and Impaired Awareness of Hypoglycemia Are Associated with Reduced Brain Gray Matter Volumes

**DOI:** 10.3389/fnins.2017.00529

**Published:** 2017-09-25

**Authors:** Petr Bednarik, Amir A. Moheet, Heidi Grohn, Anjali F. Kumar, Lynn E. Eberly, Elizabeth R. Seaquist, Silvia Mangia

**Affiliations:** ^1^Department of Radiology, Center for Magnetic Resonance Research, University of Minnesota Minneapolis, MN, United States; ^2^Department of Medicine, University of Minnesota Minneapolis, MN, United States; ^3^Division of Biostatistics, University of Minnesota Minneapolis, MN, United States

**Keywords:** structural MRI, brain volumes, type 1 diabetes, hypoglycemia, hypoglycemia unawareness

## Abstract

In this study, we retrospectively analyzed the anatomical MRI data acquired from 52 subjects with type 1 diabetes (26M/26F, 36 ± 11 years old, A1C = 7.2 ± 0.9%) and 50 age, sex and BMI frequency-matched non-diabetic controls (25M/25F, 36 ± 14 years old). The T1D group was further sub-divided based on whether subjects had normal, impaired, or indeterminate awareness of hypoglycemia (*n* = 31, 20, and 1, respectively). Our goals were to test whether the gray matter (GM) volumes of selected brain regions were associated with diabetes status as well as with the status of hypoglycemia awareness. T1D subjects were found to have slightly smaller volume of the whole cortex as compared to controls (−2.7%, *p* = 0.016), with the most affected brain region being the frontal lobe (−3.6%, *p* = 0.024). Similar differences of even larger magnitude were observed among the T1D subjects based on their hypoglycemia awareness status. Indeed, compared to the patients with normal awareness of hypoglycemia, patients with impaired awareness had smaller volume of the whole cortex (−7.9%, *p* = 0.0009), and in particular of the frontal lobe (−9.1%, *p* = 0.006), parietal lobe (−8.0%, *p* = 0.015) and temporal lobe (−8.2%, *p* = 0.009). Such differences were very similar to those observed between patients with impaired awareness and controls (−7.6%, *p* = 0.0002 in whole cortex, −9.1%, *p* = 0.0003 in frontal lobe, −7.8%, *p* = 0.002 in parietal lobe, and −6.4%, *p* = 0.019 in temporal lobe). On the other hand, patients with normal awareness did not present significant volume differences compared to controls. No group-differences were observed in the occipital lobe or in the anterior cingulate, posterior cingulate, hippocampus, and thalamus. We conclude that diabetes status is associated with a small but statistically significant reduction of the whole cortex volume, mainly in the frontal lobe. The most prominent structural effects occurred in patients with impaired awareness of hypoglycemia (IAH) as compared to those with normal awareness, perhaps due to the long-term exposure to recurrent episodes of hypoglycemia. Future studies aimed at quantifying relationships of structural outcomes with functional outcomes, with cognitive performance, as well as with parameters describing glucose variability and severity of hypoglycemia episodes, will be necessary to further understand the impact of T1D on the brain.

## Introduction

Diabetes is a growing world epidemic, with an estimated projected number of more than 800 million people living with diabetes by 2,030 (Wild et al., 2004). Diabetes is a complex metabolic disorder that affects multiple systems in the body. Type 1 diabetes (T1D) is an autoimmune disease, characterized by destruction of insulin producing cells in the pancreas resulting in absolute deficiency of insulin (Standards of Medical Care in Diabetes, 2017). Although Type 1 diabetes can manifest at any age, it is usually diagnosed in children and young adults. In comparison, type 2 diabetes is characterized by variable degrees of insulin deficiency and reduced responsiveness to insulin action. It is usually diagnosed in middle age to older people and is associated with obesity. Diabetes is characterized by hyperglycemia (high blood glucose) and over time hyperglycemia can lead to development of complications such as eye problems, nerve damage and kidney disease (Standards of Medical Care in Diabetes, 2017). Treatment with exogenous insulin is needed in people with type 1 and advanced type 2 diabetes. Treatment with insulin to tightly control glucose can reduce the risk of long term complications of diabetes but also increases risk of hypoglycemia (low blood glucose).

It has been long recognized that diabetic patients may suffer from reduced cognitive function (Miles and Root, 1922; Cukierman-Yaffe, 2014). Both type 1 and type 2 diabetes are associated with cognitive impairment and structural changes in the brain. In particular, type 1 diabetes (T1D) has been linked to performance deficits in memory, attention, information processing and executive function (Kodl and Seaquist, 2008). The neuro-structural correlates of these clinical events remain uncertain.

Identification of brain damage induced by diabetes, particularly before cognitive symptoms appear, is critical for mitigating the long-term consequences of the disease on the brain. Various non-invasive and quantitative MRI neuroimaging approaches have been utilized to objectively characterize the impact of diabetes on brain structure and function, including structural MRI, diffusion tensor imaging (DTI), magnetic resonance spectroscopy (MRS) and functional MRI (fMRI) (Moheet et al., 2015b). Subjects with T1D have been found to have reductions in both white and gray matter (GM) volumes by MRI (Musen et al., 2006; Wessels et al., 2006, 2007). They have also been shown to have lower GM density, primarily in the regions of frontal, temporal, posterior, and cerebellar regions of the brain (Musen et al., 2006; Wessels et al., 2006; Hughes et al., 2013). Moreover, in children with T1D, greater exposure to severe hypoglycemia has been associated with enlargement of hippocampal GM volume compared to children without severe hypoglycemia (Hershey et al., 2010).

In the present study we retrospectively analyzed anatomical MRI data acquired in our laboratory from a sizable number of subjects with long-standing T1D and non-diabetic subjects. The T1D group was further sub-divided in two groups based on whether subjects had normal or impaired awareness of hypoglycemia (IAH).

The typical symptoms of hypoglycemia include sweating, hunger, shakiness/tremulousness, heart pounding, and nervousness/anxiety (Tesfaye and Seaquist, 2010). These warning symptoms are mediated by activation of the nervous system and are key for recognition of hypoglycemia so that the person can take corrective actions to stop the progression of hypoglycemia. Recognition of the onset of these symptoms constitutes awareness of hypoglycemia (McAulay et al., 2001; Geddes and Frier, 2007). Recurrent exposure to iatrogenic hypoglycemia can lead to development of IAH (Cryer, 2005). IAH is estimated to occur in 20% of patients with type 1 diabetes (Geddes et al., 2008). IAH is associated with a 6-fold increased risk of developing severe hypoglycemia (an event causing such neurological changes as to require the aid of another person) (Gold et al., 1994). Questionnaires have been developed to assess awareness of hypoglycemia in people with diabetes (Gold et al., 1994; Clarke et al., 1995; Pedersen-Bjergaard et al., 2001). In this study, we primarily utilized the Cox questionnaire to categorize the status of hypoglycemia awareness in participants with diabetes (Clarke et al., 1995). The Cox questionnaire comprises 8 questions that examine the glycemic threshold at which subjects develop symptoms of hypoglycemia. This questionnaire also characterizes the subject's exposure to episodes of moderate and severe hypoglycemia. A score of four or more implies IAH, while a score of two or less reflects normal awareness of hypoglycemia. A Cox score of three however indicates an indeterminate status of awareness, in which case one can consider an additional method, such as the Gold questionnaire to further characterize impaired awareness (Gold et al., 1994). The Gold scoring method is based on the response to a single question, “Do you know when your hypos are commencing?” Results are expressed in a 7-point Likert scale (from “Always aware” to “Never aware”), where a score of four or more indicates impaired awareness.

Our goal for this study was to estimate the group-differences in structural MRI outcomes available from our datasets. Whereas, other studies have investigated differences in brain volumes between diabetic and non-diabetic subjects (van Harten et al., 2006; Moheet et al., 2015b), a distinct novel aspect of the present work was to test, within the T1D group, whether the IAH is associated with altered brain volumes. In particular, we focused on the volume of the whole cortex, separate cortical lobes, and selected cortical and sub-cortical structures, namely the anterior cingulate cortex (ACC), the posterior cingulate cortex (PCC), the hippocampus, and the thalamus. The ACC and PCC are key brain regions involved in cognition and executive function (Nachev, 2006; Leech and Sharp, 2014), while the hippocampus, a brain structure located deep in the temporal lobe, plays an essential role in learning and memory processing (Scoville and Milner, 1957; Burgess et al., 2002; Squire et al., 2004). Our specific interest in the thalamus originated from recognizing that this area may be functionally involved in the development of IAH (Mangia et al., 2012). Indeed, the thalamus is one of the brain areas which manifest increased neuronal activity during hypoglycemia as measured by increases of blood flow in healthy controls. However, such response was observed to be blunted in a group of type 1 diabetic subjects with impaired awareness to hypoglycemia (Mangia et al., 2012).

## Methods

### Subjects

Subjects were drawn from experiments that were conducted over a time-frame of 7 years from 2009 to 2016 within the context of multiple completed and on-going projects which generally aimed at describing the brain responses to hypoglycemia during hyperinsulinemic clamps (Mangia et al., 2012; Terpstra et al., 2014; Moheet et al., 2015a). Datasets that contained both T1-weighted (T1w) and T2-weighted (T2w) structural MRI of acceptable image quality as determined by visual inspection (e.g., no motion artifacts) were included in this analysis. Included T1D subjects were recruited for participation because they had T1D (defined on clinical grounds) and were between the ages of 18 and 67 years. The healthy control group was frequency matched to T1D based on age, sex, and BMI during the same time period. Recruited T1D subjects had hemoglobin A1C <8% in the 3 months before study participation, and did not have history of proliferative retinopathy or other microvascular complications. T1D subjects were also divided into two subgroups: T1D with IAH and T1D with normal awareness of hypoglycemia (NAH), as verified primarily by the Cox questionnaire (Clarke et al., 1995). In the few cases (*n* = 7) that T1D subjects had a Cox score of three (i.e., indeterminate status), we used their Gold score (Gold et al., 1994) for determining their awareness status. For 1 of these 7 patients, Gold score was missing in the database, and therefore that subject was left as “indeterminate” and excluded from the analyses focused on hypoglycemia awareness. Other exclusion criteria for both groups included history of stroke, seizures, neurosurgical procedures, or arrhythmias, use of drugs that can alter glucose metabolism (other than insulin for the T1D subjects), alcohol abuse, history of renal insufficiency with serum creatinine levels above 1.5 mg/dL, pregnancy, breastfeeding, and incompatibility with MR safety criteria.

### Protocol

During the experimental MRI session, metabolic conditions were controlled by the use of a hyperinsulinemic (2 mu/kg/min) clamp to maintain blood glucose values around 95 mg/dL (normal glycemia). Only the data collected during normal glycemia are included in this analysis. Details regarding the protocol for this insulin clamp technique have been published elsewhere (Mangia et al., 2012; Moheet et al., 2015a). The studies were carried out in accordance with the Declaration of Helsinki and with the recommendations of The Code of Federal Regulations, Institutional Review Board. The protocol was approved by the Institutional Review Board: Human Subjects Committee of the University of Minnesota. Written informed consent was obtained from all subjects prior to the experimental session.

### MRI measurements

MRI measurements were performed using either a 3 T Siemens Trio scanner (Siemens, Erlangen, Germany) or a 3 Tesla Siemens Prisma scanner (Siemens, Erlangen, Germany). Radiofrequency pulses were transmitted with the scanner body coil, while signal was received with either a 12-channels receive coil (for experiments conducted with the 3 T Trio) or a 20-channels receive coil (for experiments conducted with the 3 T Prisma). Each subject's head was carefully fixed with padding and memory foam to prevent motion artifacts while assuring that subject was comfortable with the setup. In addition, the structural T1w and T2w images were always acquired at the beginning of the study session. High resolution T1w images were acquired with a MPRAGE sequence, with the following parameters: 256 × 256 mm^2^ field of view, 160 slices, 1 mm isotropic resolution, repetition time (TR) = 2,150 ms, echo time (TE) = 2.47 ms, inversion time (TI) = 1,000 ms, with parallel acceleration factor (PAT) of 2 for a total scan time of ~5 min for each magnet. T2w scans were acquired with the T2-SPACE sequence at 1 mm isotropic resolution using the same imaging volume of the MPRAGE acquisition. For acquisitions on the 3T Trio, the T2-SPACE parameters were TR = 3,200 ms, TE = 201 ms, PAT 4, and for 3T Prisma TR = 3,200 ms, TE = 147 ms, PAT 4. Both T1w and T2w images were automatically corrected for the ${\text{B}}_{1}^{-}$ -related spatial inhomogeneity utilizing coil sensitivity profile and “prescan normalize” routine implemented in the Siemens image acquisition/reconstruction algorithm.

### Data processing

Segmentation of cortical and subcortical GM structures was performed with an identical pipeline for each subject based on Freesurfer (version 5.3, http://surfer.nmr.mgh.harvard.edu/). In the first step, the masks resulting from subcortical segmentation (aseg) (Fischl et al., 2002) and cortical parcellation (aparc) (Desikan et al., 2006) were obtained in native subject space by using T1w images. In addition, the estimated intracranial volumes (eICV), later used to adjust volumes of brain GM regions to the head size (Buckner et al., 2004), were obtained per subject. The high contrast between cerebrospinal fluid (CSF) and cerebral cortex on T2w images was utilized to refine segmentation (Glasser et al., 2013). Finally the segmentation outputs were carefully manually edited in accordance with the Freesurfer manual in several subsequent steps. The errors resulting from imperfect intensity normalization were corrected by adding control points. The remaining errors in white matter segmentation were fixed by editing of wm.mgz file. Finally, the pial surfaces were checked and edited where needed. All manual editing steps were performed by an experienced neuroradiologist (P.B.).

**Figure 1:**
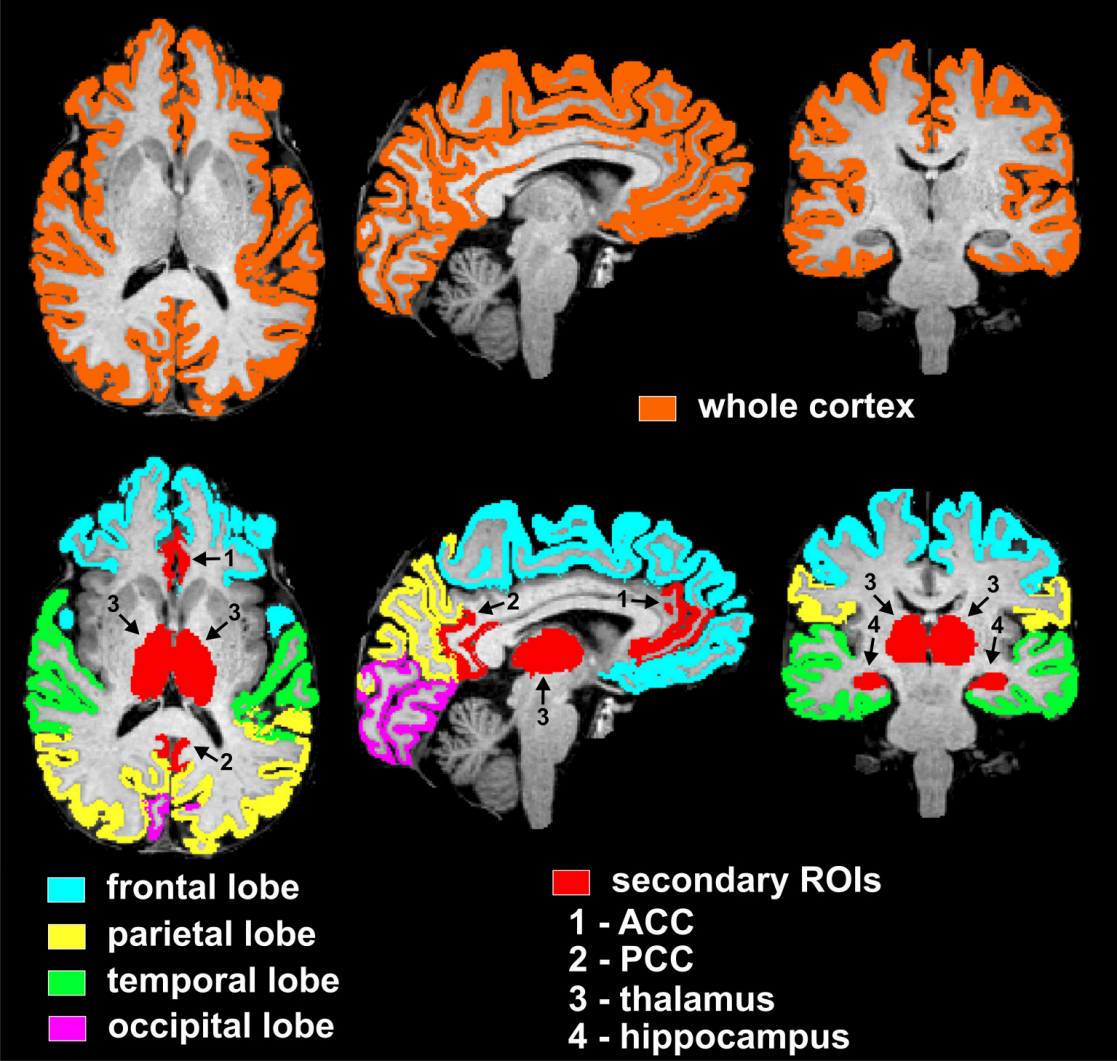
Visualization of regions of interest subjected to volumetry analyses from one representative non-diabetic subject. The top row displays the mask of the whole supratentorial cortex, which includes lobes (frontal, parietal, temporal, occipital), insula and cingulum. Bottom row depicts masks of cortical lobar regions (frontal, parietal, temporal, occipital) and secondary cortical and subcortical regions of interest (ACC, PCC, thalamus, and hippocampus). In particular, the PCC and ACC corresponded to the brain areas identified by FreeSurfer as the isthmus cingulate and rostral anterior cingulate, respectively. ROIs are here shown as combined between the two hemispheres, however the statistical analyses used both right and left hemisphere data points per ROI per subject.

The selected nine cortical and subcortical regions of interest (ROIs) are listed in **Tables 2–4**, and are shown in Figure 1. In particular, the PCC corresponded to the brain areas identified by FreeSurfer as the isthmus cingulate, while the ACC corresponded to the rostral anterior cingulate. Volumes were calculated from each ROI separately for right and left hemisphere, so that two data points were obtained per subject per ROI. Volumes of the segmented brain structures were scaled (i.e., divided) by eICV per subject.

Finally, the operator performing the manual editing was blinded to the hypoglycemia awareness status of the T1D subjects, but was un-blinded to the diabetes status (controls or T1D). In order to assess the possible bias induced by the partial un-blinding of the operator, differences in the whole cortex volumes obtained before and after manual edits were calculated per subject, with positive numbers indicating volume reductions after editing.

### Statistical analyses

Subject age, BMI, A1c, and disease duration were summarized with means and standard deviations (SD) and compared between groups using linear models; sex and scanning protocol distribution were compared across groups using Fisher's exact test. For each of the nine ROIs separately, volumes (left and right hemispheres combined) were summarized by group using means and standard deviations. Group comparisons of GM volumes per ROI were tested (1) between all T1D subjects and controls, (2) between T1D with NAH and T1D with IAH, and (3) between controls and each of T1D with NAH and T1D with IAH. For such comparisons, linear mixed models were used that adjusted for age, sex, BMI, hemisphere, and protocol. For the NAH vs. IAH comparisons within the T1D group, we fit models with and without additional correction for duration of disease and A1c. Our primary test of interest was for the whole cortex. Within the whole cortex, false discovery rate (FDR) (Benjamini and Hochberg, 1995) correction was used for multiple testing of the 4 lobes, and separately for multiple testing of the additional 4 cortical and subcortical ROIs (namely the ACC, PCC, hippocampus, and thalamus). When comparing controls to each of the groups of T1D subjects with NAH and with IAH, the FDR correction included the additional *p*-value correction for those two pairwise comparisons. Statistical significance results are presented both without and with the multiple-testing corrections. Finally, hemisphere by group interactions were evaluated (but were left out of the final models since they were non-significant for all ROIs), and differences in the whole cortex volumes obtained before and after manual edits were compared with unpaired two-tailed *t*-test between the controls and T1D subjects.

## Results

A total of 102 datasets (50 from non-diabetic controls and 52 from T1D subjects) were evaluated. Subjects were evenly distributed among females and males in both control and T1D groups (Table 1). T1D subjects had good glycemic control, with an average A1C of 7.2%, and their disease duration was 20 ± 11 years on average. Both T1D and control groups were 36 years old on average (*p* = 0.86), while the T1D-NAH group was on average 11 years younger than the T1D-IAH group (*p* = 0.0005). T1D-IAH subjects also tended to have slightly lower A1C as compared to the T1D-NAH group (*p* = 0.042). No group-differences in BMI were observed. Image quality was high/excellent for all datasets, and no differences were observed among groups or between imaging protocols. In addition, on average, 0.04 ± 1.5% and 0.06 ± 1.1% of voxels were removed by the operator in controls and T1D subjects, respectively (*p* = 0.92).

**Table 1:** Subject characteristics.

**Group**	***n***	**Age (years)**	**Sex (M/F)**	**BMI (kg/m^2^)**	**T1D duration (years)**	**A1C (%)**	**Protocol (Trio/Prisma)**
Control	50	36 ± 14	25/25	25 ± 4	–	–	18/32
T1D	52	36 ± 11	26/26	26 ± 4	20 ± 11	7.2 ± 0.9	19/33
*p*-values^*^		0.86	0.99	0.25	–	–	0.99
T1D-NAH	31	32 ± 10	18/13	26 ± 4	17 ± 10	7.4 ± 0.8	10/21
T1D-IAH	20	43 ± 10	8/12	27 ± 5	25 ± 11	6.9 ± 0.8	9/11
*p*-values^§^		0.0005	0.26	0.45	0.008	0.042	0.39

* *Comparisons between control and T1D subjects*.

§ *Comparisons between T1D-NAH and T1D-IAH subjects*.

The GM volume of the whole cortex was slightly smaller for T1D subjects than controls (by −2.7%, *p* = 0.016). Among the 4 lobes, the frontal cortex was the most affected (by −3.6%, *p* = 0.024, corrected), whereas only a trend of smaller volume was observed for the parietal lobe (by −2.9%, *p* = 0.081, corrected). No differences were observed when considering the other ROIs (Table 2).

**Table 2:** Volumetry comparisons between T1D subjects and controls.

**Region**	**Group values (mean ± *SD*)**		**Group-comparisons**		
	**Control (*n* = 50)**	**T1D (*n* = 52)**	Diff^*^ (%)	**unadj_p**	**fdr_p**
Whole cortex	0.33 ± 0.02	0.32 ± 0.02	−**2.7**	0.016	0.016
Frontal lobe	0.119 ± 0.010	0.115 ± 0.009	−**3.6**	0.006	0.024
Occipital lobe	0.031 ± 0.003	0.030 ± 0.002	−3.4	0.084	0.112
Parietal lobe	0.079 ± 0.006	0.077 ± 0.006	−2.9	0.040	0.081
Temporal lobe	0.073 ± 0.005	0.072 ± 0.005	−1.3	0.345	0.345
ACC	0.0033 ± 0.0004	0.0033 ± 0.0004	−0.3	0.976	0.976
Hippocampus	0.0058 ± 0.0005	0.0058 ± 0.0005	0.0	0.800	0.976
PCC	0.0033 ± 0.0004	0.0032 ± 0.0004	−2.7	0.256	0.512
Thalamus	0.0104 ± 0.0009	0.0101 ± 0.0010	−2.6	0.131	0.512

*Linear mixed models used for the comparisons included age, sex, BMI, hemisphere and protocol as covariates. False discovery rate correction was applied for the 4 lobes, and, separately, for the ACC, hippocampus, PCC, and thalamus. Shaded areas indicate p < 0.05, and bold numbers indicate group differences for which fdr_p < 0.05. ACC (anterior cingulate cortex), PCC (posterior cingulate cortex), unadj_p (unadjusted p-value), fdr_p (p-value after false discovery rate correction)*.

* *Differences were calculated as T1D minus Control*.

When comparing IAH vs. NAH in T1D subjects (Table 3), the GM volume of the whole cortex was smaller by an even larger extent (namely −7.9%, *p* = 0.0009) than what was observed between T1D subjects and controls. Smaller volumes were observed in particular in the frontal lobe (−9.1%, *p* = 0.006, corrected), parietal lobe (−8.0%, *p* = 0.015, corrected), and temporal lobe (−8.2%, *p* = 0.009, corrected). Such observations were not driven by differences in T1D duration and A1C among groups, since these models adjusting for T1D disease duration and A1C showed similar or even stronger statistical significance than when comparisons were not corrected for those parameters (Table 3). No volume differences in the other ROIs were observed.

**Table 3:** Volumetry comparisons for T1D subjects between those with NAH and IAH.

**Region**	**Group values (mean ± *SD*)**		**Group-comparisons**				
				**Without adjustment for T1D duration and A1C**		**With adjustment for T1D duration and A1C**	
	**T1D with NAH (*n* = 31)**	**T1D with IAH (*n* = 20)**	Diff^*^ (%)	**unadj_p**	**fdr_p**	**unadj_p**	**fdr_p**
Whole cortex	0.33 ± 0.01	0.30 ± 0.02	−**7.9**	0.002	0.002	0.0009	0.0009
Frontal lobe	0.120 ± 0.007	0.108 ± 0.008	−**9.1**	0.002	0.008	0.002	0.006
Occipital lobe	0.031 ± 0.002	0.029 ± 0.003	−3.8	0.593	0.593	0.342	0.342
Parietal lobe	0.079 ± 0.005	0.073 ± 0.006	−**8.0**	0.013	0.018	0.011	0.015
Temporal lobe	0.074 ± 0.004	0.069 ± 0.005	−**8.2**	0.008	0.016	0.005	0.009
ACC	0.0034 ± 0.0004	0.0032 ± 0.0005	−5.4	0.528	0.704	0.377	0.528
Hippocampus	0.0059 ± 0.0004	0.0057 ± 0.0006	−3.3	0.478	0.704	0.396	0.528
PCC	0.0033 ± 0.0004	0.0032 ± 0.0004	−2.7	0.966	0.966	0.876	0.876
Thalamus	0.0104 ± 0.0008	0.0097 ± 0.0011	−5.2	0.313	0.704	0.292	0.528

*Linear mixed models used for the comparisons included age, sex, BMI, hemisphere and protocol as covariates, both without and with additional adjustment for disease duration and A1c. False discovery rate correction was applied for the 4 lobes, and, separately, for the ACC, hippocampus, PCC, and thalamus. Shaded areas indicate p < 0.05, and bold numbers indicate group differences for which fdr_p < 0.05. ACC (anterior cingulate cortex), PCC (posterior cingulate cortex), unadj_p (unadjusted p-value), fdr_p (p-value after false discovery rate correction)*.

* *Differences were calculated as IAH minus NAH for the T1D subjects*.

Differences observed between T1D subjects with and without impaired awareness were very similar to those observed between T1D with IAH and controls (Table 4), namely −7.6% in whole cortex (*p* = 0.0002), −9.1% in frontal lobe (*p* = 0.0003, corrected), −7.8% in parietal lobe (*p* = 0.002, corrected), and −6.4% in temporal lobe (*p* = 0.019, corrected). On the other hand, T1D with NAH did not present significant volume differences as compared to controls in any ROI.

**Table 4:** Volumetry comparisons of controls with T1D-NAH and T1D-IAH subjects.

**Region**	**Group-comparisons**					
	**T1D-NAH vs. Controls**			**T1D-IAH vs. Controls**		
	Diff^*^ (%)	**unadj_p**	**fdr_p**	Diff^*^ (%)	**unadj_p**	**fdr_p**
Whole cortex	0.4	0.632	0.632	−**7.6**	0.0001	0.0002
Frontal lobe	0.0	0.435	0.569	−**9.1**	0.00004	0.0003
Occipital lobe	−2.0	0.207	0.331	−5.7	0.118	0.237
Parietal lobe	−0.1	0.719	0.719	−**7.8**	0.0006	0.002
Temporal lobe	1.9	0.498	0.569	−**6.4**	0.007	0.019
ACC	−1.8	0.812	0.812	−3.7	0.794	0.812
Hippocampus	1.5	0.507	0.812	−2.0	0.810	0.812
PCC	1.4	0.255	0.812	−4.6	0.468	0.812
Thalamus	−0.6	0.371	0.812	−5.8	0.108	0.812

*Linear mixed models used for the comparisons included age, sex, BMI, hemisphere and protocol as covariates. False discovery rate correction was applied for the 4 lobes and two pairwise comparisons, and, separately, for the ACC, hippocampus, PCC, thalamus and the two pairwise comparisons. Shaded areas indicate p-values < 0.05, and bold numbers indicate group differences for which fdr_p < 0.05. ACC (anterior cingulate cortex), PCC (posterior cingulate cortex), unadj_p (unadjusted p-value), fdr_p (p-value after false discovery rate correction)*.

* *Differences were calculated as T1D-NAH or T1D-IAH subjects minus controls*.

Finally, no hemisphere by group interaction was observed for any ROI. Also, the two protocols were not found to impact the volumetric outcomes.

## Discussion

This cross-sectional morphometric MRI study examined the association of T1D with GM volumes. In this study, we found that subjects with long standing T1D had smaller whole cortex and frontal lobe GM volumes compared to non-diabetic controls of similar age, sex and BMI. In this study, subjects with diabetes had good glycemic control based on A1C and did not have any severe microvascular complications. We also noted a trend toward T1D having smaller GM volume in the parietal region. Overall the magnitude of these differences was small, namely 3.6% in the frontal lobe and 2.7% in the whole cortex. However, structural effects of larger magnitude were present in T1D patients with IAH as compared to patients with NAH (e.g., 7.9 and 9.1% in whole cortex and frontal lobe, respectively), as well as compared to controls (e.g., 7.6 and 9.1% in whole cortex and frontal lobe, respectively). Interestingly, patients with NAH did not show volume reductions as compared to controls. These observations are novel because association between IAH and reduced brain volumes in patients with T1D diabetes has never been reported before.

The exact mechanisms underlying development of IAH are not known and could be related to alterations in both brain hypoglycemia sensing and impaired coordination of the counter-regulatory response. Alterations in neurotransmission, upregulation of brain glucose transport or availability of alternate fuels like lactate for brain metabolism (as a result of exposure to recurrent hypoglycemia) may contribute to development of IAH (Tesfaye and Seaquist, 2010). Strict avoidance of hypoglycemia may partially restore awareness of hypoglycemia (Dagogo-Jack et al., 1994; Fanelli et al., 1994; Leelarathna et al., 2013), however this is very challenging for insulin treated patients with diabetes to maintain long-term.

Importantly, our comparisons took into account age differences between the two patient populations (T1D subjects with IAH tend to be older than T1D subjects with NAH), along with differences in A1C (T1D subjects with IAH tend to have slightly lower A1C than T1D subjects with NAH) and differences in diabetes duration (T1D subjects with IAH tend to have longer disease duration than T1D subjects with NAH). Therefore, based on our results, we can rule out that our findings were driven by age or simply by disease duration. On the other hand, since T1D patients who develop IAH are generally those who experience episodes of hypoglycemia more frequently, one can speculate that the long-term exposure to recurrent hypoglycemia may be associated with reduced brain volumes. To test such hypothesis, future investigations should include objective measures of antecedent hypoglycemia episodes by means of continuous glucose monitoring.

Smaller brain volumes between T1D subjects and non-diabetic subjects are generally consistent with previous published literature. Musen et al. reported reduced GM density in the region of left and right superior temporal gyri, left angular gyrus, left middle temporal and middle frontal gyri, and left thalamus in subjects with T1D relative to control subjects (Musen et al., 2006). Another study reported reduced GM volume in the frontal lobe in subjects with T1D compared to controls (Hughes et al., 2013). Smaller GM volumes in T1D have been linked to poor glycemic control, severe hypoglycemia and presence of severe microvascular complications (Moheet et al., 2015b).

In people with Type 2 diabetes, several studies have shown evidence of hippocampal atrophy and reduced performance on neurocognitive testing (den Heijer et al., 2003; Bruehl et al., 2009). In our study we did not see significant differences in hippocampal volumes of subjects with T1D compared to controls. These findings are consistent with previous small studies which also did not find evidence of hippocampal atrophy in subjects with long standing T1D (Lobnig et al., 2006; Bednarik et al., 2015). Interestingly, whereas group differences were observed in the frontal and parietal lobes, our analysis did not reveal volume differences in two sub-regions of such lobes that constitute the major hubs of the default mode network, namely the ACC and PCC. Diabetes or hypoglycemia awareness status did not impact the thalamic volume either, despite this region has been suggested to be functionally involved in the development of hypoglycemia unawareness based on the blunted thalamic responses to hypoglycemia observed in IAH-T1D subjects as compared to non-diabetic controls (Mangia et al., 2012). These findings remind to apply general caution when anticipating structural group differences of a brain area based on functional group differences observed in that area.

MRI studies, including the present one, usually involve only limited numbers of subjects. Therefore, it remains a challenge to coherently describe how the many contributors such as age, diabetes duration, glucose control (i.e., A1C levels) and glucose variability, among others, mediate the impact of diabetes on the brain especially when analyses are performed at the voxel level, which is inherently more demanding in terms of multiple comparison corrections. To partially overcome such a challenge, in this study we chose a morphometry analysis based on estimating the volumes of aggregated large brain areas that were mostly automatically segmented by FreeSurfer routines and, most importantly, are maintained in the native space of the subject. Such an ROI-based approach allows a straightforward normalization of brain volumes based on the total intra-cranial volume of the subject and does not require excellent alignment of the normalized brains as is necessary for voxel-wise comparisons. Also, the use of automatic Freesurfer segmentation, and standardized operator routines to correct segmentation outcomes, allowed identifying the regions of interest with minimized operator biases. In addition, a distinct strength of our study design was the excellent matching in the frequency of age, sex, and BMI between T1D and non-diabetic subjects.

One obvious limitation of this study is its cross-sectional design. We do not have longitudinal A1C data or information about previous episodes of severe hypoglycemia, so we cannot assess the relationship of exposure to hyper- and hypoglycemia on GM volume. Another limitation of our study is that we did not perform neurocognitive testing in these subjects and cannot assess if these subtle reductions in GM volumes were associated with decline in cognitive function.

## Conclusions

We conclude that T1D is associated with a small but significant reduction of the whole cortex GM volume, mainly in the frontal lobe. Similar structural effects occur when comparing, within the T1D subjects, patients with IAH vs. those with normal awareness. The clinical significance of these subtle changes in GM volume is not clear. Future studies are needed to examine if these subtle reductions in GM volumes are associated with cognitive impairment.

## Author contributions

PB and AM have contributed equally to the work. PB participated in acquisition and analysis of the data, and in preparing the manuscript. AM participated in acquisition and interpretation of the data, and preparing the manuscript. HG and AK participated in acquisition of the data, and editing the manuscript. LE participated in design of the work, analysis and interpretation of the data, and in editing the manuscript. ES participated in design of the work, interpretation of the data, and editing the manuscript. SM participated in design of the work, acquisition, analysis, interpretation of the data, and preparing the manuscript.

### Conflict of interest statement

The authors declare that the research was conducted in the absence of any commercial or financial relationships that could be construed as a potential conflict of interest.
